# Fracture and Growth Are Competing Forces Determining the Fate of Conformers in Tau Fibril Populations*

**DOI:** 10.1074/jbc.M116.715557

**Published:** 2016-04-14

**Authors:** Virginia Meyer, Michael R. Holden, Hilary A. Weismiller, Gareth R. Eaton, Sandra S. Eaton, Martin Margittai

**Affiliations:** From the Department of Chemistry and Biochemistry, University of Denver, Denver, Colorado 80208

**Keywords:** aggregation, Alzheimer disease, amyloid, electron paramagnetic resonance (EPR), fibril, prion, Tau protein (Tau)

## Abstract

Tau fibrils are pathological aggregates that can transfer between neurons and then recruit soluble Tau monomers by template-assisted conversion. The propagation of different fibril polymorphs is thought to be a contributing factor to phenotypic diversity in Alzheimer disease and other Tauopathies. We found that a homogeneous population of Tau fibrils composed of the truncated version K18 (residues 244–372) gradually converted to a new set of fibril conformers when subjected to multiple cycles of seeding and growth. Using double electron-electron resonance (DEER) spectroscopy, we observed that the distances between spin labels at positions 311 and 328 in the fibril core progressively decreased. The findings were corroborated by changes in turbidity, morphology, and protease sensitivity. Fibrils that were initially formed under stirring conditions exhibited an increased fragility compared with fibrils formed quiescently after multiple cycles of seeding. The quiescently formed fibrils were marked by accelerated growth. The difference in fragility and growth between the different conformers explains how the change in incubation condition could lead to the amplification of a minor subpopulation of fibrils. Under quiescent conditions where fibril breakage is minimal, faster growing fibrils have a selective advantage. The findings are of general importance as they suggest that changes in selective pressures during fibril propagation in the human brain could result in the emergence of new fibril conformers with varied clinicopathological consequences.

## Introduction

Fibrillar inclusions composed of the microtubule-associated protein Tau are a pathological hallmark of Alzheimer disease and various other fatal neurodegenerative disorders, collectively referred to as Tauopathies (1–3). Tau pathology often progresses in a temporal and spatial manner that is characteristic for a particular Tauopathy (4–6). Variations from “standard” pathology are also known, representing subtypes of disease (7, 8). Recent evidence suggests that the progression or “spreading” of Tau pathology could have its origin in the transfer of Tau aggregates from one cell to another (9–12) and the subsequent recruitment of naïve Tau monomers onto the fibril ends (13). This mechanism of propagation is remarkably similar to the propagation of prions (14–17), with template-assisted conversion serving as the driving force for conformational change (18–20). An intriguing phenomenon of prion diseases is the existence of different strains, phenotypic variations in the disease that may be linked to conformationally distinct prion aggregates (21–23). Similar relationships between protein structure and disease phenotype have been identified for Tau, where aggregates from different Tauopathies induced distinct inclusions in cell culture and mouse brain (24, 25). The structural characteristics of the Tau aggregates could be propagated through multiple generations, suggesting clonal properties of the fibrils (25). From the prion field, it is known that when sequences between recruited protein and protein template are different, strains may switch (26). Strain switching may also occur when the prion proteins have identical primary structure, but are exposed to different host environments (27), suggesting that specific changes in growth conditions can result in the emergence of novel protein conformers. Whether changes in growth conditions can have similar effects on the structure of Tau fibrils is unknown. Such understanding, however, is important, as variable neuronal environments in the human brain could contribute to phenotypic diversity.

The co-expression of six different Tau isoforms in the adult human brain adds to the complexity of Tau pathologies. Tau isoforms range in size from 352 to 441 amino acids and differ by the presence or absence of a 29 or 58 residue insert in the N-terminal half and by the inclusion or exclusion of the second of four microtubule binding repeats in the C-terminal half. Each of the repeats contains 31–32 residues. Based on the number of repeats, Tau isoforms may be grouped into 3-repeat (3R)^3^ Tau and 4-repeat (4R) Tau. The repeat region forms the protease resistant core of the fibrils (28–30) and is surrounded by a largely disordered, fuzzy coat made of the N- and C-terminal flanking regions (31). The core of Tau fibrils assumes a cross-β structure in which β-strands extend perpendicular to the long fibril axis and are separated ∼4.7 Å apart (32, 33). Additionally, strands within individual sheets of the fibrils are arranged parallel and in-register (34–36).

Monomeric Tau is intrinsically disordered (37, 38). Long-range contacts between the flanking regions and the microtubule binding repeats (39–41) prevent homotypic interactions. As a consequence, truncated versions of Tau that contain only the repeat region exhibit greatly accelerated aggregation kinetics (42). Two such constructs are K18 and K19 (43). The constructs span residues 244 to 372 (based on the numbering of the largest isoform) and represent the 4R Tau and 3R Tau isoforms, respectively. K18 and K19 have served as widely used proxies of their full-length counterparts, exhibiting similar properties with respect to seeding (44), uptake (45), and transmission (46).

To gain insights into the core of Tau fibrils beyond strand-registry, we recently introduced pairs of spin labels into the third repeat of K18 and K19 and measured the distances between them (47) using a technique called double electron-electron resonance (DEER) spectroscopy (48, 49). As these distances are intramolecular in nature, they provide information on the structural relationships within different parts of the same protein. A similar approach has recently been used to gain insights into the fibril structures of α-synuclein (50, 51) and islet amyloid polypeptide (52). We identified key differences in the conformations of K18 and K19. Most noticeable, K19 fibrils were structurally homogeneous whereas K18 fibrils were not (47). The heterogeneity of K18 fibrils was unexpected, as they had passed through multiple cycles of seeding and growth. The heterogeneity offers a unique opportunity of investigating the relative stability of individual Tau fibril conformers and their structural evolution. Using the multicycle seeding scheme in conjunction with careful structural analysis, we observe that a specific change in reaction conditions markedly alters the ensemble of K18 fibrils, whereas K19 fibrils remain largely unaltered. The changes in K18 structure can be explained by different selective pressures under altered growth conditions. The findings have important implications for the propagation of Tau fibrils in the human brain.

## Experimental Procedures

### 

#### 

##### Constructs

K18 and K19 with native cysteines replaced by serines (positions 291 and 322 in K18; position 322 in K19) were cloned into pET28b. For simplicity, these constructs are referred to as K18 and K19 throughout the text. The double cysteine mutants K18 311/328 and K19 311/322 (native cysteines replaced by serines) were generated by site-directed mutagenesis. The synthesis and cloning of these four constructs were described previously (35, 47).

##### Protein Expression and Purification

All plasmids were transformed into the *Escherichia coli* strain BL21 (DE3). Single colonies were transferred into Miller LB medium and grown at 37 °C for 17 h under agitation. The cultures were diluted 1:100 into fresh medium and incubated at 37 °C with agitation until *A*_600_ ∼0.8. Expression was induced through addition of 1 mm isopropyl-β-d-thiogalactopyranoside and continued for 4 h at 37 °C. Bacterial pellets were collected by centrifugation at 3,000 × *g* for 20 min and were resuspended in buffer (20 mm PIPES, 500 mm NaCl, 1 mm EDTA, and 50 mm β-mercaptoethanol, pH 6.5). Cells were stored at −80 °C prior to purification. Samples were incubated at 80 °C for 30 min, precipitating most bacterial proteins. Bacteria were then sonicated for 1 min using a sonic dismembrator (D100 series, Fisher Scientific) set to 50% power. Cellular debris was separated by centrifugation at 15,000 × *g* for 30 min. The supernatant, which contained soluble protein, was added to 55% *w*/*v* ammonium sulfate. The mixture was rocked at 22 °C for 1 h, and precipitated Tau was collected by centrifugation at 15,000 × *g* for 10 min. Pellets were resuspended in 2 mm dithiothreitol (DTT/water), sonicated for 40 s at 50% power, and passed through 0.45 μm Acrodisc GxF/GHP filters (Pall Life Sciences). The sample was loaded onto a Mono S cation exchange column (GE Healthcare) and eluted with a linear salt gradient. Fractions containing the highest concentrations of protein were pooled and loaded onto a Superdex 200 gel filtration column (GE Healthcare), eluting with time. Protein-containing fractions were again pooled and Tau was precipitated overnight at 4 °C through addition of a 3-fold volumetric excess of 5 mm DTT/acetone. Purified protein pellets were collected by centrifugation at 15,000 × *g* and stored in 2 mm DTT/acetone at −80 °C.

##### Protein Solubilization and Spin-labeling

Protein pellets were dissolved in 8 m guanidine hydrochloride. The K18 311/328 and K19 311/322 double cysteine mutants were spin-labeled through addition of a 10-fold molar excess of 1-oxyl-2,2,5,5-tetramethyl-Δ3-pyrroline-3-methyl)methane-thiosulfonate (MTSL, Toronto Research Chemicals) followed by 1 h of incubation at 22 °C. Unreacted spin label and denaturant were removed by passing the samples over PD-10 columns (GE Healthcare). K18 and K19 were processed in the same manner with the exception that the labeling step was omitted. The elution buffer contained 100 mm NaCl and 10 mm HEPES at pH 7.4. This buffer was used for all following reactions. Protein concentrations were determined by BCA assay (Pierce).

##### Fibril Assembly and Multicycle Seeding

Fibrils were assembled by mixing 25 μm Tau (K18 or K19) with a 2-fold molar excess of heparin (average MW = 4400, Celsius, EN-3225) and continuously stirring the sample (1,400 μl) with a teflon-coated micro stir bar (5 × 2 mm) at 160 rpm for 3 days at 22 °C. To produce seeds, the samples (500 μl) were sonicated for 20 s on ice using a microtip probe (2 mm diameter) connected to a sonic dismembrator set at 20% power. These seeds were used to initiate the first elongation reaction, referred to as cycle 1. In this step, 25 μm Tau (K18 or K19) and 50 μm heparin were combined with 10% seeds (monomer equivalents), and fibrils grew quiescently for 1 h at 37 °C. The samples were cooled for 10 min on ice and sonicated as above to produce seeds for the next elongation reaction. The procedure was repeated up to 15 times, resulting in a series of fibrils that differed in the number of seeding cycles. For turbidity measurements (below), fibrils from this series were analyzed directly. For all other experiments (EPR, EM, proteolysis, cross-seeding, fibril fragility, and growth), fibrils from select cycles in the series were removed, sonicated as above, combined with Tau monomers, elongated, and then measured. Specifically, 5% seeds (monomer equivalents) were mixed with 50 μm Tau (K18 or K19) and 12.5 μm heparin and incubated for 14 h at 37 °C. For DEER experiments (below), Tau monomers in the elongation reaction were a mixture of 49 μm K18 and 1 μm spin-labeled K18 311/328 or 49 μm K19 and 1 μm spin-labeled K19 311/322.

##### DEER Sample Preparation, Measurement, and Data Analysis

Fibrils were pelleted by centrifugation at 130,000 × *g* for 30 min. Pellets were resuspended in 10–20 μl of fresh buffer and transferred into 1.6-mm outer diameter (o.d.) quartz Q-band EPR tubes (VitroCom). Prior to freezing, continuous wave (CW) spectra were collected at X-band to check that spin label had been incorporated within the fibrils. The 1.6-mm o.d. Q-band tube was supported in a 4-mm o.d. X-band tube, and samples were analyzed on a Bruker EMX spectrometer with an ER 4119HS resonator at 25 °C. All spectra were collected at 20 milliwatt with 100 kHz modulation frequency and 3.0 G modulation. The scan width was 150 G. After CW data collection, the samples were flash frozen in liquid N_2_ and stored at −80 °C until DEER measurement. DEER was performed at Q-band on a Bruker ELEXSYS E580 spectrometer with an ER 5107D2 dielectric resonator. An E580–400U ELDOR unit was used for the second microwave source in conjunction with a SuperQ-FT bridge. An Oxford CF935 cryostat was used for measurements at 80 K. Data were collected using four microwave pulses; the pump pulse was positioned on the center frequency of the resonator Q-dip, corresponding to the maximum height of a field-swept echo-detected spectrum, and the observe pulses were positioned 37 MHz below the pump frequency. At the observe frequency, π/2 = 38–44 ns and at the pump frequency, π = 40 ns. Pulse delays and additional parameters have been described previously (47, 53). All DEER data were analyzed with DEER Analysis 2011 (54) using Tikhonov regularization.

##### Turbidity

Turbidity was measured in a WinCary Bio100 UV-vis spectrophotometer (Agilent) using quartz cuvettes. The instrument baseline was corrected using buffer. The OD_340_ was collected for fibrils at each cycle in the multicycle seeding process. The monomer (25 μm K18 or K19) and heparin (50 μm) controls produced negligible signal. All data were collected in triplicate.

##### Proteolysis

Fibrils from cycles 1 and 10 (see above) were mixed with proteinase K (PK) (7 nm and 70 nm for K18; 14 nm and 140 nm for K19) at a total volume of 100 μl and incubated for 1 h at 22 °C. A buffer-adjusted sample (in the absence of protease) served as a control. Proteolysis was terminated by addition of protease inhibitor, phenylmethylsulfonyl fluoride (Sigma), to a final concentration of 4 mm. The degree of proteolysis was assessed by SDS-PAGE and Coomassie Blue staining utilizing 4–20% gradient gels.

##### Limited Fibril Fracture

K18 fibrils (100 μl of 50 μm monomer equivalents) from cycles 1 and 10 were fractured for 60 s in a bath sonicator (QSONICA) set to 5% power and 22 °C. The samples were then diluted to 10 μm and analyzed by EM (below).

##### Elongation Kinetics

K18 fibrils (500 μl of 50 μm monomer equivalents) from cycles 1 and 10 were sonicated for 2 min on ice with a tip (2 mm diameter) directly immersed in the sample using a sonic dismembrator set to 20% power. The seeds were sedimented for 30 min at 130,000 × *g* and then resuspended in buffer. Protein concentrations were determined using the BCA assay (Pierce). Samples were diluted to 10 μm and analyzed by EM. Fibril growth was assessed by thioflavin T fluorescence (55, 56) using a Tecan Infinite M1000 plate reader. Specifically, 25 μm K18 monomers were mixed with 50 μm heparin and 5 μm thioflavin T. The mixtures were equilibrated to 25 °C in 96-well polystyrene plates. The reactions were initiated by addition of 2% seeds (monomer equivalents), and the final reaction volumes were 200 μl. The samples were excited at 440 nm. Emission was measured every 60 s at 480 nm. All reactions were carried out six times, and the results were averaged.

##### Seeding Barrier

Tau monomers (25 μm K18 or K19) were mixed with 50 μm heparin and 5% seeds (produced by 2 min sonication of cycle 1 and cycle 10 fibrils), and allowed to incubate for 24 h at 37 °C in capped polyallomer tubes (1.5 ml). The fibrils were sedimented for 30 min at 130,000 × *g*. The pellets and supernatants were adjusted to equal volumes with SDS sample buffer. Equivalent amounts of samples were analyzed by SDS-PAGE (15% gels) and Coomassie Blue staining.

##### Transmission Electron Microscopy (EM)

250-mesh carbon coated copper grids were placed for 60 s onto 10 μl of sample droplets containing Tau fibrils (10 μm). Thereafter, liquid was removed and grids placed for 60 s onto 10 μl droplets of 2% uranyl acetate. The grids were air-dried on filter paper and images recorded with a Philips/FEI Tecnai-12 transmission electron microscope at 80 keV equipped with a Gatan CCD camera.

## Results

### 

#### 

##### Tau Fibril Populations Evolve during Consecutive Cycles of Seeding and Growth

The ability to form distinct fibril conformers from proteins with identical sequence and to perpetuate these conformers by template-assisted conversion is a unique property of amyloids (57). Structural polymorphism requires that the recruited proteins exhibit a high degree of plasticity as they are molded into different conformational states. Tau proteins possess such plasticity (55, 58). We recently observed that K18 fibrils, when subjected to 5 cycles of seeding and growth, formed a heterogeneous mixture of conformers (47). Since the conformers compete for the same pool of Tau monomers we now wanted to explore the relative stabilities of these species. Would the distribution of fibril conformers change if the number of seeding cycles was altered? And if so, what would be the molecular mechanism?

**FIGURE 1.**
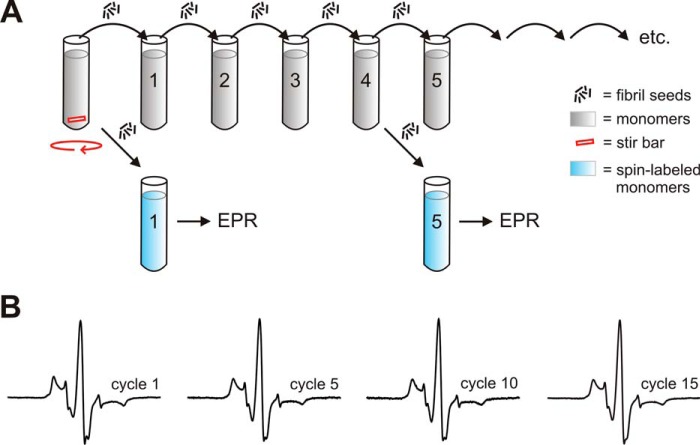
**Structural analysis of Tau fibrils by CW EPR.** *A*, schematic of multicycle seeding reaction. K18 monomers are mixed with a 2-fold molar excess of heparin and allowed to form fibrils for 3 days while stirring. After sonication, the fibril seeds are mixed with fresh K18 monomers to produce a new generation of fibrils referred to as cycle 1. The procedure is repeated up to 15 times. In these templated reactions, fibrils are grown quiescently. At distinct steps, seeds are removed and used to form an independent set of fibrils for structural analysis. These fibrils have 2% spin-labeled K18 311/328 incorporated into their structure and are analyzed by EPR. The dilution is necessary to avoid spin-spin interactions along the fibril axis. *B*, CW EPR spectra of K18 fibrils from cycles 1, 5, 10, and 15 collected at a scan width of 150 G. The spectra are the first derivatives of the corresponding absorption spectra. The separation of outer peaks (67 G) indicates that the spin labels are immobilized in the fibril core.

A schematic of the experimental procedure is depicted in Fig. 1*A*. In a first step, K18 fibrils were formed for 3 days under agitating conditions by stirring. These fibrils were then sonicated and used as seeds for the next generation of fibrils (cycle 1). The procedure was repeated through 15 cycles. Each cycle involved the transfer of 10% fibril seeds (monomer equivalents) into a new tube containing K18 monomers (25 μm) followed by 60 min quiescent incubation at 37 °C. The production of successive generations of fibrils served as a starting point for structural analysis by electron paramagnetic resonance (EPR), a technique that can measure the distances between paramagnetic centers (59). We reasoned that incorporation of doubly spin-labeled Tau monomers into the different fibril ensembles could report on the relative distributions of conformers. For this purpose K18 was labeled with the nitroxide spin label MTSL (60) at positions 311 and 328, sites that are nestled in the fibril core (34, 35). The protein was then mixed with a 50-fold molar excess of unlabeled K18 (total protein concentration = 50 μm), added to 5% seeds (monomer equivalents), and allowed to grow for 14 h at 37 °C. The dilution with unlabeled protein was necessary to prevent intermolecular interactions between spin labels along the long fibril axis. EPR-active samples were produced for cycles 1, 5, 10, and 15. In a first set of measurements, we tested whether spin-labeled Tau monomers were properly incorporated into the fibrils. For this purpose, fibrils were sedimented and analyzed by CW EPR spectroscopy. The separation between outer peaks for all spectra was 67 G (Fig. 1*B*). This value is typical for immobilized spin labels and indicates full incorporation of the proteins into the fibrils (61, 62). Furthermore, the absences of spectral distortions that are characteristic for labels separated less than 2.0 nm (63, 64) suggest that the spin-labeled proteins were not preferentially stacked on top of each other, but instead distributed throughout the fibril.

**FIGURE 2.**
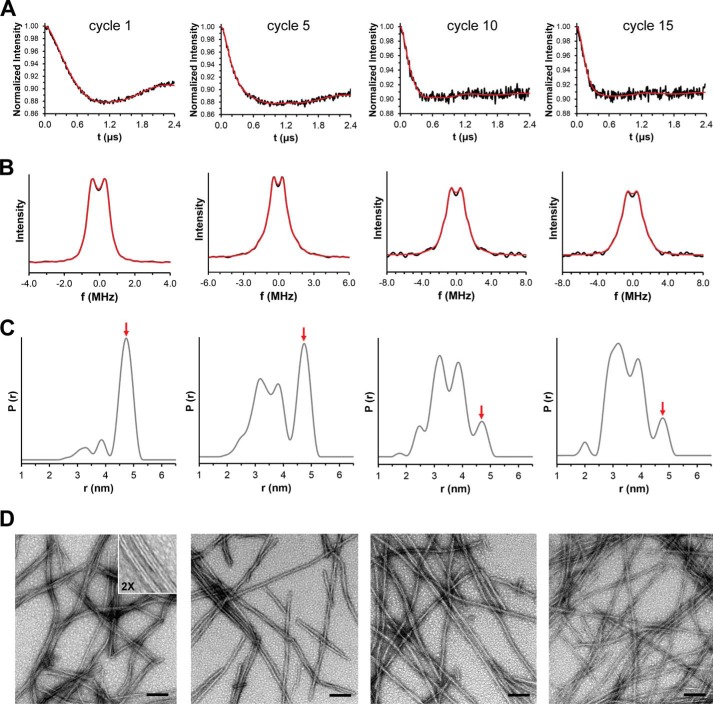
**Structural evolution of K18 fibrils monitored by DEER and EM.** DEER data were collected for Tau fibrils following different cycles of seeding. Data are analyzed by Tikhonov regularization. Background subtracted dipolar evolution curves in the time domain (*A*) and frequency domain (*B*) shown as *black traces* with best fits in *red. C*, distance distributions. The *red arrow* highlights changes of the fibril conformer at 4.8 nm. *D*, EM analysis of K18 fibrils. Cycle 1 fibrils have a distinct striated ribbon appearance with varying numbers of parallel filaments (diameter per filament = 7–8 nm), highlighted in the *inset*. Fibrils at later cycles are dominated by a typical twisted appearance (diameter ≈ 14 nm, helix periodicity = 90–180 nm). Scale bars, 100 nm. Panels from *left to right* represent data for fibrils from cycle 1, cycle 5, cycle 10, and cycle 15, respectively.

In a next set of measurements, the intramolecular distances between the spin labels at positions 311 and 328 were determined by DEER spectroscopy. Specifically, dipolar evolution traces were collected using the four-pulse sequence (65) and fit by Tikhonov regularization in the time and frequency domains (Fig. 2, *A* and *B*). Remarkably, the resulting distance distributions changed for subsequent seeding cycles (Fig. 2*C*). Whereas cycle 5 produced a similar distance distribution to the one that was previously reported for this number of cycles (47), other cycles had markedly different outcomes. Surprisingly, the most homogeneous population was observed for fibrils formed in the first cycle, which showed a dominant peak at 4.8 nm. As additional cycles were applied to the seeds, the conformations characterized by distances at 3.2 and 3.8 nm were enriched. The population of the 4.8 nm conformation was decreased relative to the other conformations (Fig. 2*C*, *red arrow*). These trends appeared to reach an end point at approximately cycle 10, as cycle 15 resulted in similar distance distributions and dipolar evolution curves (Fig. 2, *A* and *C*). Whether the minor peaks observed at 2.5 nm for cycle 10 and 2.0 nm for cycle 15 represent novel fibril conformers or whether they are artifacts of the data analysis is currently unknown. Also, the number of fibril conformers is unlikely limited to three. First, different conformers could be characterized by the same distances. Secondly, other parts of the protein, which are not monitored here, may assume different structures (66). Electron microscopic analysis of the fibrils formed in the different seeding cycles revealed that cycle 1 fibrils were morphologically distinct from those formed in later cycles (Fig. 2*D*). The fibrils in cycle 1 had a predominantly striated ribbon appearance with different degrees of lateral associations. In contrast, the majority of fibrils formed in later cycles were twisted.

**FIGURE 3.**
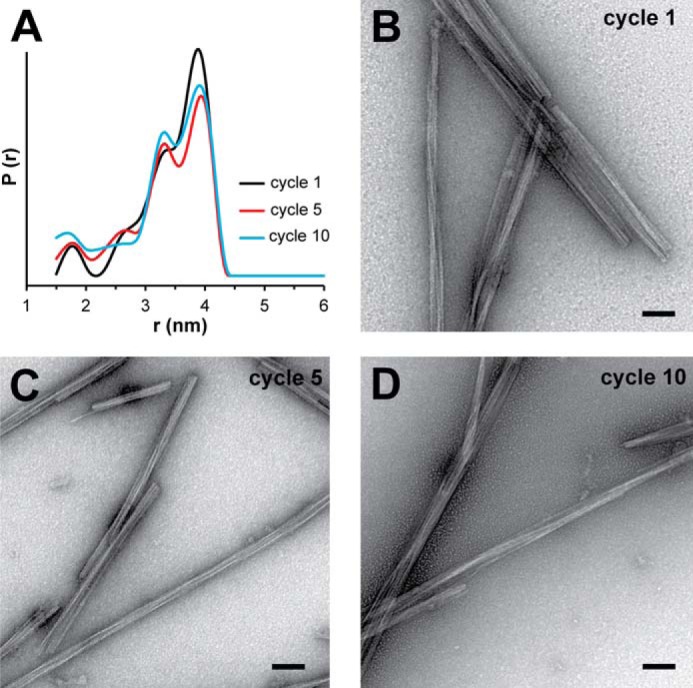
**Conformational stability of K19 fibrils revealed by DEER and EM.** *A*, distance distributions for K19 fibrils collected after cycle 1 (*black trace*), cycle 5 (*red trace*), and cycle 10 (*blue trace*). In parallel, fibrils were stained with uranyl acetate and analyzed by transmission electron microscopy. Electron micrographs of fibrils from cycle 1 (*B*), cycle 5 (*C*), and cycle 10 (*D*). Scale bars, 100 nm. All fibrils exhibit the same ribbon-like morphology with different degrees of lateral associations. Individual filaments within these assemblies have a diameter of 7–8 nm.

In a next set of experiments we tested whether K19 fibrils would undergo similar structural evolution. For this purpose, K19 fibrils were formed in an analogous manner as K18 fibrils (see above) and then subjected to consecutive cycles of seeding and growth. Since the spin labeled 311/328 construct of K19 does not produce any measurable distances when incorporated into the fibrils (presumably the labels are separated >5.0 nm apart and thus cannot be detected by DEER (47)) we chose the 311/322 construct instead. When the distance distributions between fibrils from cycles 1, 5, and 10 were compared, only minor changes in populations were observed. The distance distributions were characterized by a major peak at 3.9 nm and a minor peak at 3.4 nm (Fig. 3*A*). The results suggest that these fibrils are more resistant toward conformational change. The findings were further supported by EM analysis, which revealed unaltered morphologies for fibrils from different cycles (Fig. 3, *B–D*). Interestingly, the K19 fibrils had ribbon-like features similar to those of K18 fibrils in cycle 1.

##### Different Tau Fibril Populations Have Distinct Physical Properties

**FIGURE 4.**
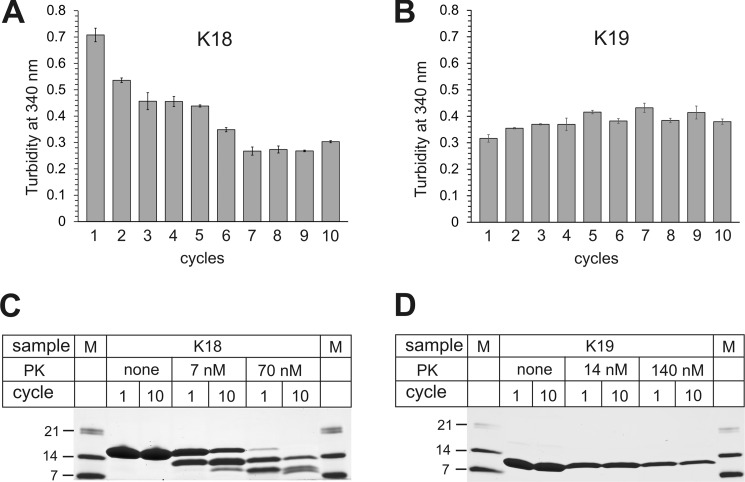
**Structural analysis of K18 and K19 fibrils by turbidity and proteinase K digestion.** The turbidity of K18 fibrils (*A*) and K19 fibrils (*B*) from different cycles was measured at 340 nm. All values represent average ± STD (*n* = 3 experiments). The decreased turbidity of K18 fibrils in later cycles agrees with changes in the fibril population. No such changes are observed for K19 fibrils. K18 fibrils (*C*) and K19 fibrils (*D*) from cycle 1 and cycle 10 (25 μm, monomer equivalents) were proteolyzed for 1 h at 22 °C with equal amounts of proteinase K (*PK*). The samples were analyzed by SDS-PAGE and Coomassie Blue staining. *M*, molecular weight marker. K18 fibrils from cycle 10 were more sensitive to degradation than fibrils from cycle 1. No differences in protease sensitivity were observed for K19 fibrils.

During DEER sample preparation, differences in physical properties of the K18 fibrils were observed. Prior to centrifugation, the solutions of cycle 1 fibrils in buffer were turbid, whereas those of cycles 10 and 15 were significantly more transparent. To quantify these differences, the degree of turbidity for each seeding cycle was assessed by light scattering. Fig. 4*A* shows a decrease in scattering for fibrils formed after an increasing number of seeding cycles. Differences between fibrils were more pronounced following centrifugation. Cycle 1 pellets were opaque white and tightly packed. Cycle 10 pellets were transparent, gelatinous and loosely packed. Cycle 5 pellets displayed intermediate characteristics. The effect of variation in packing efficiency is also apparent from differences in the signal to noise ratio of the EPR data (Fig. 2*A*). K19 fibrils showed no differences in packing. Accordingly, these fibrils exhibited similar scattering throughout consecutive cycles (Fig. 4*B*).

We next examined whether the fibrils exhibited varying degrees of protease resistance. K18 and K19 fibrils from cycles 1 and 10 were subjected to proteolysis using incremental concentrations of PK. Fig. 4*C* shows solubilized K18 fibrils analyzed by SDS-PAGE following incubation. Without addition of protease, fibrils from cycles 1 and 10 did not degrade and were shown to be the same concentration. Following one hour of incubation at 37 °C, cycle 10 fibrils displayed more rapid degradation than cycle 1 fibrils at both PK concentrations. No such differences in degradation were observed for K19 fibrils (Fig. 4*D*), supporting the conclusion that the structures of these fibrils remained largely unchanged over multiple seeding cycles. All experiments thus far (DEER distance measurements, EM analysis, turbidity tests, and PK sensitivity) indicate that the K18 (but not K19) fibril populations change during consecutive cycles of seeding. The underlying cause for this structural change, however, is not clear from these experiments. Importantly, it is not related to temperature since K18 fibrils formed at 37 °C produced the same distance distribution as fibrils formed at 22 °C (data not shown).

##### Differences in Fragility and Growth Can Explain Changes in Fibril Structure

**FIGURE 5.**
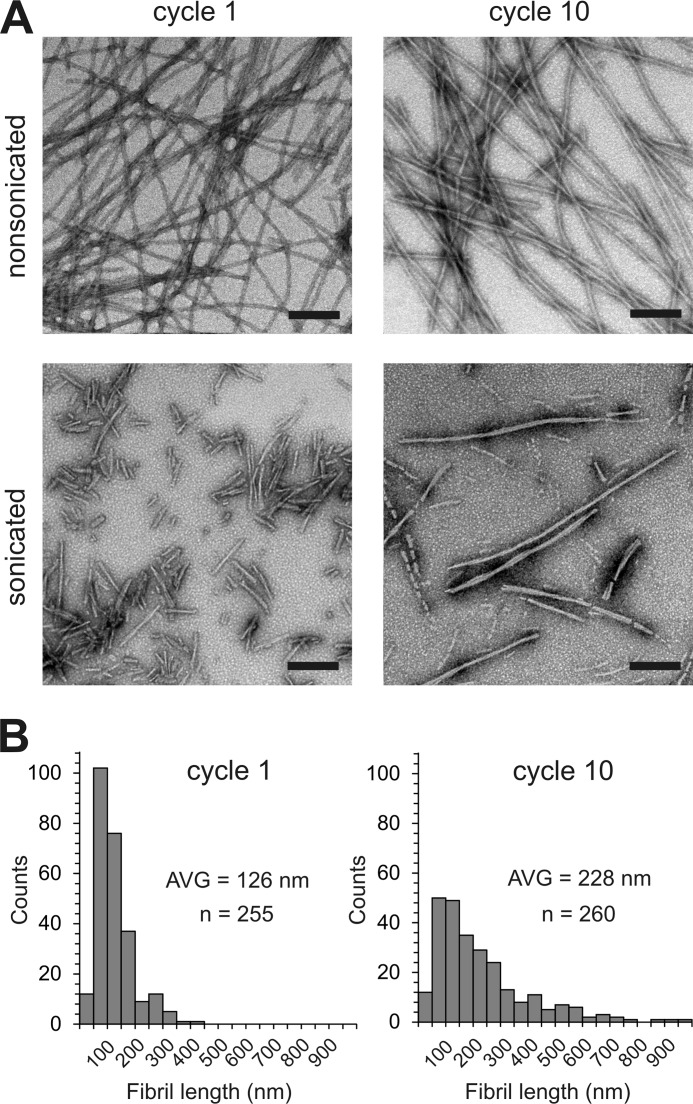
**K18 fibril fragilities determined using EM.** *A*, cycle 1 and cycle 10 fibrils are analyzed by negative stain EM before (upper panels) and after sonication (*lower panels*). The fibrils are fractured under mild conditions in a bath sonicator. Scale bars, 200 nm. When subjected to identical stress, cycle 1 fibrils (diameter per filament = 7–8 nm) are distinctly shorter than cycle 10 fibrils (diameter ≈ 14 nm). *B*, length distributions for sonicated fibrils from cycle 1 (*left panel*) and cycle 10 (*right panel*). The average fibril lengths are 126 and 228 nm, respectively. *AVG*, average. The results indicate that cycle 1 fibrils are more fragile than cycle 10 fibrils.

In the next set of experiments we wanted to elucidate the molecular mechanism for the observed evolution in K18 fibril structure. An important detail of the aggregation protocol (Fig. 1*A*) is that the original fibrils, which served as seeds for cycle 1, were formed while stirring. In contrast, fibrils that were utilized as seeds in all consecutive cycles were grown quiescently. The question arose whether this change in growth condition could be responsible for the gradual shift in fibril populations. We hypothesized that differences in fragilities and growth rates between fibrils could lead to the selective amplification of distinct conformers under the altered conditions. To test this hypothesis, fibrils from cycles 1 and 10 were subjected to low intensity sonication. Specifically, tubes containing 100 μl of the samples (50 μm K18) were placed into a bath sonicator and exposed for 1 min to continuous pulsing at 5% power. Non-sonicated fibrils served as controls. Negative stain EM images revealed that sonicated cycle 1 fibrils were distinctly shorter than cycle 10 fibrils (Fig. 5), suggesting that the former fibrils had a greater tendency to break.

**FIGURE 6.**
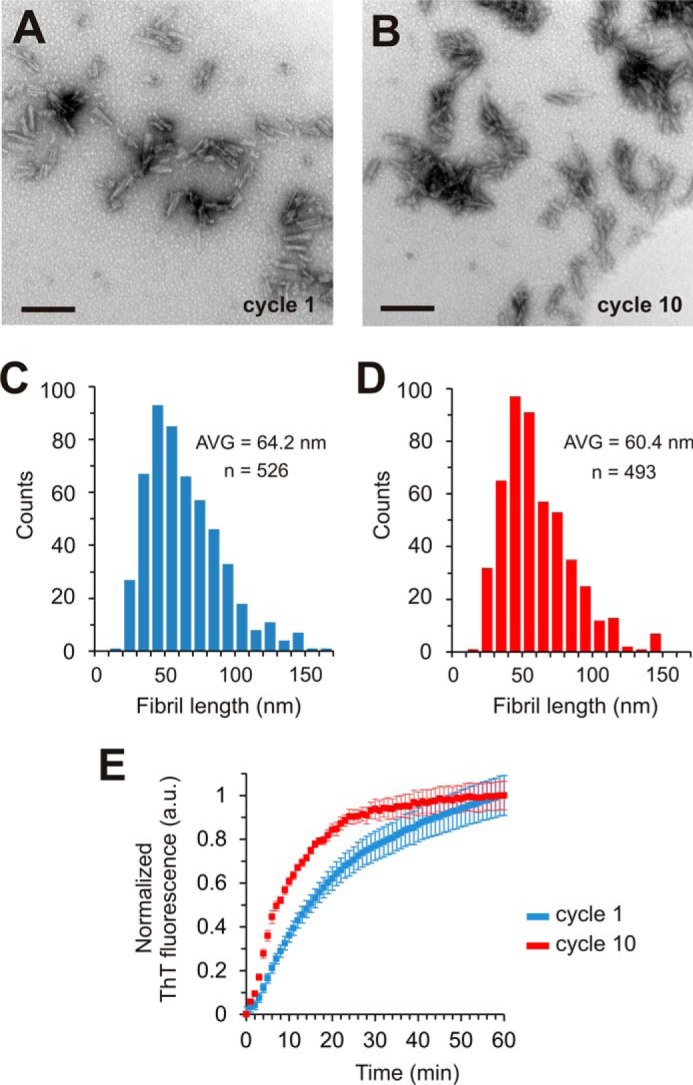
**Analysis of Tau fibril growth rates.** Negative stain EM images of cycle 1 (*A*) and cycle 10 (*B*) fibrils subjected to harsh sonication conditions with the sonicator tip immersed in the fibril solution. Scale bars, 200 nm. Length distributions of cycle 1 (*C*) and cycle 10 (*D*) fibrils. The lengths of 526 fibrils from cycle 1 and 493 fibrils from cycle 10 were measured using Image-J. The average lengths of cycle 1 and cycle 10 fibrils are 64 nm and 60 nm, respectively. *AVG*, average. *E*, 2% seeds (monomer equivalents) were mixed with 25 μm K18 monomers and 50 μm heparin. Fibril growth of cycle 1 fibrils (blue trace) and cycle 10 fibrils (*red trace*) was monitored by thioflavin T fluorescence. All values represent average ± S.E. (*n* = 6 experiments). The data indicate that cycle 10 fibrils elongate faster than cycle 1 fibrils.

Next, we investigated the growth rates of the two different fibril types. For this purpose, cycle 1 and cycle 10 fibrils were sonicated extensively (for 2 min at 20% power) on ice with a 2-mm diameter tip directly immersed into the samples. The fractured fibrils were then visualized by negative stain EM. Very short fibril fragments were observed in both cases (Fig. 6, *A* and *B*). The lengths of the fibrils from 10 different grids were measured using Image-J (67). Both types of fibrils had similar length distributions (Fig. 6, *C* and *D*). The seeds were sedimented by ultracentrifugation and equal concentrations of seeds (2% monomer equivalents) were mixed with 25 μm K18 monomers to initiate fibril growth. The reactions were monitored using thioflavin T fluorescence. Strikingly, seeds produced from cycle 10 fibrils resulted in fibril growth that was more than twice as fast as from seeds produced from cycle 1 fibrils (Fig. 6*E*), with *t*_50_ values of 8 min and 17 min, respectively. The same trend was consistently observed with three independent batches of seeds. Collectively, the results demonstrate that cycle 1 and cycle 10 fibrils differ in their fragilities and growth rates providing a molecular explanation for the evolution of conformers as fibrils are propagated through multiple cycles of seeding and growth.

##### Seeding Barrier between 3R and 4R Tau Is Polymorph-independent

**FIGURE 7.**
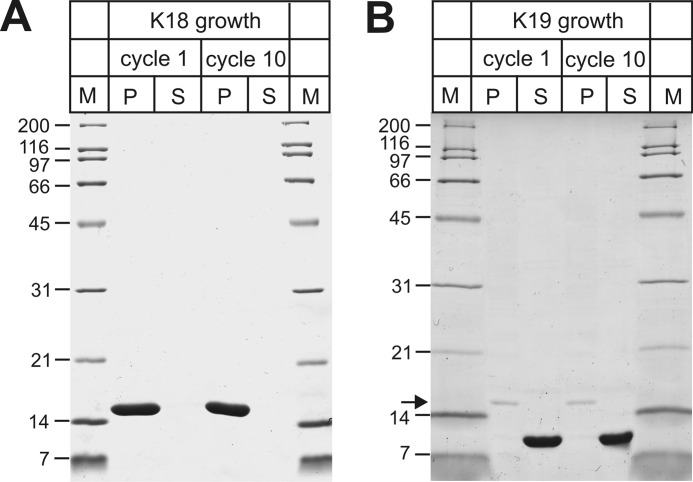
**Examination of Tau seeding barrier for distinct conformations.** 5% seeds (monomer equivalents) from cycle 1 and cycle 10 fibrils were mixed with 25 μm K18 or K19 monomers and 50 μm heparin. Fibrils were allowed to grow for 24 h at 37 °C. Equivalent amounts of pellets (*P*) and supernatants (*S*) were analyzed by SDS-PAGE and Coomassie Blue staining. Whereas K18 monomers grow onto K18 seeds (*A*), K19 monomers do not (*B*). The *arrow* in *B* refers to K18 protein bands that originate from the seeds. The data highlight that both populations of K18 fibril conformers are unable to recruit K19 monomers.

In the past we had identified a seeding barrier that prevented 3R Tau monomers from growing onto 4R Tau seeds (55). Remarkably, this barrier was overcome when 4R Tau monomers were grown onto 3R Tau seeds. This suggested that 4R Tau monomers possess a high degree of plasticity and that the barrier is determined by the conformation of the seeds. Here we tested the seeding properties of cycle 1 and cycle 10 K18 (4R) fibrils with respect to K19 (3R) growth. For this purpose, 5% seeds (monomer equivalents) were mixed with 25 μm Tau and allowed to grow for 24 h under quiescent conditions. The samples were then sedimented by ultracentrifugation. Equal amounts of pellets and supernatants were analyzed by SDS-PAGE and Coomassie Blue staining. As expected K18 monomers efficiently grew onto the K18 seeds (Fig. 7*A*). K19 monomers, however, remained in the supernatant regardless of whether cycle 1 or cycle 10 K18 fibrils were used for seeding (Fig. 7*B*). The results reveal the existence of a seeding barrier for both fibril populations highlighting the incompatibility of these conformers with K19 growth. The findings are in agreement with previous observations, which suggested that only extended conformers of K18, marked by long spin separations between labels at positions 311 and 328 (>5.0 nm), are able to effectively recruit K19 monomers (47). The conformers tested here (before and after population shift) are characterized by shorter spin separations (<5.0 nm, Fig. 2*C*).

## Discussion

Different conformers of Tau fibrils are thought to play distinct roles in determining pathology and disease phenotype in human Tauopathies (24, 25). Fibril populations that spread throughout the brain pass through repetitive cycles of breakage, transfer, and growth. Little is known regarding whether fibril structure is preserved in this process. Such understanding, however, is important, as shifts in fibril populations could contribute to phenotypic diversity. Using precisely defined *in vitro* conditions, we set out to study the stability of Tau fibril populations through multiple cycles of seeding and growth. We observe that, when stirred, 4R Tau monomers form homogeneous fibrils with striated ribbon morphology. The number of lateral associations in these fibrils varies. However, the molecular structure is preserved, as only a single distance (∼4.8 nm) is observed between spin labels at positions 311 and 328. When these fibrils are subjected to intense shearing followed by quiescent growth, the population of fibril conformers changes. This change is gradual with short distance conformers (3.2 and 3.8 nm separation between spin labels) becoming the dominant species after repetitive cycles of amplification. The change coincides with a shift in fibril morphology toward twisted forms. Differences in turbidity, proteolysis, growth, and fracture further corroborate that the original Tau fibrils and the fibrils that emerge after multiple cycles of seeding are structurally distinct. In contrast, only minor variations in fibril populations are observed, when 3R Tau is propagated through multiple cycles of seeding, suggesting that these fibrils are more resistant to structural change. Previously we had demonstrated that sequence mismatches between 4R Tau templates and 4R Tau monomers can cause shifts in fibril populations based on conformational incompatibilities (53). In the current study, the sequences of 4R Tau template and 4R Tau monomer are the same. The change in fibril structure is clearly a result of altered growth conditions, which change from stirring to quiescent.

**FIGURE 8.**
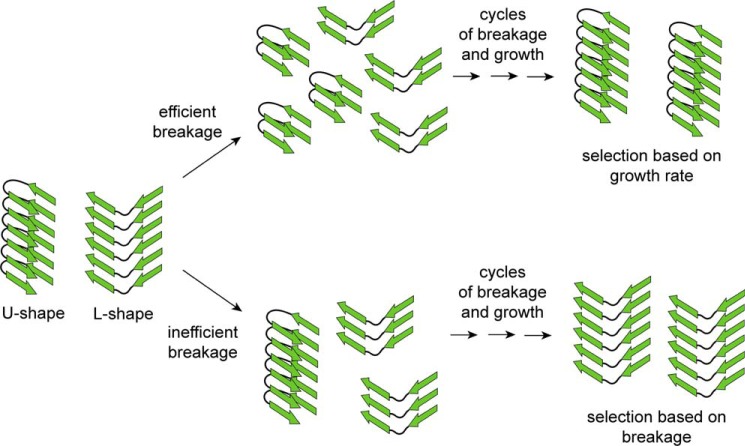
**Conformational selection based on fracture and growth.** In a heterogeneous mixture of Tau fibril conformers, depicted as stacks of β-stranded segments (*green arrows*) in U- and L-shape conformations, efficient breakage (intense sonication) results in seeds with identical extensions along the fibril axis. Under these conditions the number of fibril ends that can recruit soluble monomers is the same for the different conformers giving the faster growing seed (U-shape) a selective advantage. Over repetitive cycles of fracture and growth this conformer will become the dominant species (upper reaction path). Inefficient breakage selectively fractures the more fragile conformer (L-shape). If repeated over multiple cycles (continuous stirring) this conformer will become the dominant species (lower reaction path). Note that the depicted conformers are only models. The real structures of Tau fibrils will be more complex and include additional β-sheets. The number of cycles required for evolving a dominant species will depend on the structural composition of the original ensemble and the specific parameters of fracture and growth. In cases where the fragilities and growth rates of different conformers are similar the populations may have multiple dominant fibril species. It is conceivable that within neuronal cells, molecular chaperones or other machineries could facilitate the breakage of fibrils and influence growth.

If the structure of the fibril template is faithfully imprinted onto the newly recruited Tau monomers, a subpopulation of fibril conformers must exist in the original population that is selectively amplified under the changed conditions. Interestingly, the existence of such quasispecies has been postulated in the emergence of new prion strains (68, 69), although errors in imprinting could add to structural diversification (70). Remarkably, the same three strains of K18 fibrils coexist in different series of cycling experiments (47, 53), suggesting that these strains possess a high probability of formation. A key difference between the conformers of Tau fibrils in the original and evolved populations lies in their fragilities and growth rates. Tau fibrils that are produced under mechanical stress (stirring) have increased fragilities, but decreased growth rates. The production of new fibril ends during fracture offers fragile fibrils a selective advantage over more stable, albeit faster growing fibrils. Once this selective pressure is removed, *i.e.* fibrils are grown quiescently, conformers evolve toward faster growing species. It is important to notice that each seeding cycle involves a sonication step. This means that even fibrils that are grown quiescently undergo a fracturing step at the beginning of the cycle. However, the forces acting on these fibrils are different from the forces acting on fibrils formed during stirring. This sets in motion an alternate path of fibril selection (Fig. 8). Intense mechanical stress (tip sonication) efficiently breaks all fibrils regardless of conformation, producing seeds with identical lengths. Similar results were previously observed for two different fibril conformers of α-synuclein, which were sonicated for prolonged periods of time (71). Under these conditions, faster growing fibril conformers will have a selective advantage. Mild mechanical stress (stirring or bath sonication) causes inefficient fibril breakage. More fragile conformers will break more rapidly giving a selective advantage to these fibrils. The subtle balance between fracture and growth determines the fate of conformers within the population. Given the right conditions, different conformers may be populated to similar degrees.

In the case of the yeast prion protein Sup35, it was demonstrated that more brittle fibrils caused stronger phenotypes, as these fibrils were more successful in being segregated into daughter cells (72). Here, the chaperone Hsp104, a remodeling factor of Sup35 (73, 74) plays a critical role in facilitating breakage (75, 76). However, the dosage of Hsp104 is also important as elevated concentrations appear to dissolve the fibrils and thereby terminate propagation (75, 76). An equivalent chaperone system does not exist in metazoans. Hence, it is not clear how Tau fibrils are broken into smaller seeds. Such breakage, however, is necessary for propagation, as only short fibrils are taken up by cells (77). Interestingly, a powerful disaggregase system involving Hsc70, Hsp110, and the class B J-protein DNAJB1 has recently been identified for Parkinson's related α-synuclein fibrils (78). The chaperone system works by effectively breaking and depolymerizing the fibrils. It is conceivable that similar machinery could fracture and depolymerize Tau fibrils, although other mechanisms might operate as well.

The experiments presented in this study were performed in a minimalistic environment utilizing a truncated version of 4R Tau. The molecular environment within the human brain where Tau fibrils propagate far exceeds the complexity of the herein investigated system. For one, different Tau isoforms co-exist in the same cell. Nevertheless, the findings suggest that the efficiency of fibril breakage and the competency of growth, both of which may be modulated by cell-specific concentrations of chaperones, could affect the selection of Tau fibril conformers. Other conformation-dependent mechanisms such as fibril clearance, cellular uptake, protein interactions with the fibril surface, and isoform recruitment may add to the selection process. If Tau pathology is propagated by templated conversion, different fibril conformers will compete for a limited pool of monomers. It is tempting to speculate that the stochastic nature of fibrillization, paired with differences in genetic background and variations in cellular environments, could influence the specific path of Tau fibril spreading and its clinicopathological consequences.

## Author Contributions

M. M. designed the research. V. M., M. R. H., and H. A. W. performed the experiments. G. R. E. and S. S. E. provided technical advice and comments on the manuscript. M. M. and V. M. wrote the paper. All authors reviewed the results and approved the final version of the manuscript.

## Footnotes

3 The abbreviations used are:

3R: 3-repeat
EPR: electron paramagnetic resonance
DEER: double electron-electron resonance
4R: 4-repeat
PK: proteinase K.
